# Data-Driven User-Type Clustering of a Physical Activity Promotion App: Usage Data Analysis Study

**DOI:** 10.2196/30149

**Published:** 2022-08-01

**Authors:** Christina Kranzinger, Verena Venek, Harald Rieser, Sonja Jungreitmayr, Susanne Ring-Dimitriou

**Affiliations:** 1 Salzburg Research Forschungsgesellschaft mbH Salzburg Austria; 2 Department of Sport and Exercise Science University of Salzburg Salzburg Austria

**Keywords:** active and assisted living, app usage, cluster analysis, Jenks natural breaks algorithm, Partitioning Around Medoids algorithm, physical activity promotion, usage groups

## Abstract

**Background:**

Physical inactivity remains a leading risk factor for mortality worldwide. Owing to increasing sedentary behavior (activities in a reclining, seated, or lying position with low-energy expenditures), vehicle-based transport, and insufficient physical workload, the prevalence of physical activity decreases significantly with age. To promote sufficient levels of participation in physical activities, the research prototype *Fit-mit-ILSE* was developed with the goal of making adults aged ≥55 years physically fit and fit for the use of assistive technologies. The system combines active and assisted living technologies and smart services in the *ILSE* app.

**Objective:**

The clustering of health and fitness app user types, especially in the context of active and assisted living projects, has been mainly defined by experts through 1D cluster thresholds based on app usage frequency. We aimed to investigate and present data-driven methods for clustering app user types and to identify usage patterns based on the *ILSE* app function *Fit at home*.

**Methods:**

During the 2 phases of the field trials, *ILSE* app log data were collected from 165 participants. Using this data set, 2 data-driven approaches were applied for clustering to group app users who were similar to each other. First, the common approach of user-type clustering based on expert-defined thresholds was replaced by a data-driven derivation of the cluster thresholds using the Jenks natural breaks algorithm. Second, a multidimensional clustering approach using the Partitioning Around Medoids algorithm was explored to consider the detailed app usage pattern data.

**Results:**

Applying the Jenks clustering algorithm to the mean usage per day and clustering the users into 4 groups showed that most of the users (63/165, 38.2%) used the *Fit at home* function between once a week and every second day. More men were in the *low* usage group than women. In addition, the younger users were more often identified as *moderate* or *high* users than the older users, who were mainly classified as *low* users; moreover, the regional differences between Vienna and Salzburg were identified. In addition, the multidimensional approach identified 4 different user groups that differed mainly in terms of time of use, gender, and region. Overall, the younger women living in Salzburg were the users with highest average app usage.

**Conclusions:**

The application of different clustering approaches showed that data-driven calculations of user groups can complement expert-based definitions, provide objective thresholds for the analysis of app usage data, and identify groups that can be targeted individually based on their specific group characteristics.

## Introduction

### Background

Around 1.65 million people ≥65 years old were living in Austria in 2018. This number will increase to 2.5 million in 2040 according to the 2018 population forecast of the Austrian Conference on Spatial Planning [1]. The increasing number of older adults highlights the need for health-promoting measures to prevent immobility and ensure a good quality of life for this growing segment of society.

To date, the recommended physical activity dosage for healthy insufficiently active adults consists of 150 minutes of moderate to vigorous physical activity per week [2,3]. In addition, according to the World Health Organization (WHO) [4], people ≥65 years old with poor mobility should be physically active for ≥3 days per week, focusing on functional balance to prevent falls. However, every exercise session, whether short (seconds to minutes) or long (minutes to hours), counts toward reducing the risk of cardiometabolic morbidity and mortality [5].

To promote physical activity, the fitness program *Fit-mit-ILSE* was introduced as part of the Austrian Active and Assisted Living (AAL) research project, *fit4AAL*. The project aimed to make the increasingly technology-familiar generation of ≥55 years fit and *fit for the use* of assistive technologies. Within the program, an app called *ILSE* was developed to support and encourage the older adult population to exercise at home or outdoors through videos and courses on a tablet or 3D camera system.

In particular, the analysis of app usage is of interest in determining the acceptance of apps in general and fitness apps. Actual app usage can be monitored by analyzing the questionnaire or app usage log data, which may include exercise frequency tracking. App usage and reasons for fitness and health apps have been addressed in various studies. For example, the study by Dadaczynski [6] analyzed the user experience and the actual use of the web-based intervention *Healingo Fit* with a user experience questionnaire and usage data. The results showed particularly high satisfaction values for the dimensions of attractiveness, stimulation, and originality. Analysis of the log data revealed that the app was visited on average 65 times a day within 6 weeks [6]. The study by Schneider et al [7] discussed the use of the AAL prototype CARIMO and found from the analysis of the log data that the use of the CARIMO app remained quite stable during the test months. The study by Meyer et al [8] analyzed the use of activity trackers and described their general use, changes over time, and characteristic patterns of activity tracker usage in the long term.

The definition of app usage groups characterizes users and can be used as a grouping variable in statistical tests. From an exercise prescription perspective, identifying user types is important for creating a personalized fitness training program. Such personalization aims to engage in health-promoting physical activity, improve physical fitness levels, and maintain users’ exercise fidelity [9]. A wrongly tailored exercise program, that is, if the exercises are too difficult and too intense, will not be adopted by the user [9,10]. By analyzing app usage and identifying the types of usage, app limitations can be identified. For example, it is possible to investigate which user groups are using the app and which groups may not yet be addressed and therefore may need other engagement strategies.

Thus far, different approaches have been used to identify and group app usage groups. Meyer et al [8] and Schneider et al [7] used expert or predefined categorization limits to define different use patterns or user types. Data-driven clustering of users was performed by Lim et al [11], who used daily step counts from activity trackers of 140,000 individuals and clustered them into 16 user segments.

However, to the best of our knowledge, no common definition exists for classifying fitness app users into user groups. Groups of app users can be clustered based on either expert knowledge [7,8] or data-driven methods [11], which offers a less subjective grouping option and can help experts identify an objective classification.

### Objective

This paper proposes 2 data-driven usage-type clustering approaches that can be used to characterize the users of the training module of the ILSE program. We aimed to answer the following 2 research questions: How can *ILSE* app user types be defined in a data-driven manner? How are these user types characterized?

Log data from the *ILSE* app’s *Fit at Home* training module were used for the analysis, and users were categorized in 2 different ways. The 1D clustering approach was based on usage frequency, and the classes were defined using the Jenks natural breaks (Jenks NB) [12] algorithm, with the number of groups and the lower bound set manually. The second multidimensional clustering approach was based on multiple features of app usage, and the Partitioning Around Medoids (PAM) [13] clustering algorithm was applied to identify app usage patterns.

## Methods

### Overview

The analysis was based on *ILSE* app usage data from the AAL project *fit4AAL*. The ILSE system combines smart home components and smart services such as the *ILSE* fitness app. The fitness app aimed to encourage and support elderly exercise at home or outdoors via prerecorded videos and courses on a tablet or 3D camera computer. Participants in the program used the *ILSE* app in a field study for several weeks. There were 2 field trial phases in the Austrian cities of Vienna and Salzburg as well as in the surrounding area of Salzburg. The participants were able to use the system for 13 consecutive weeks before they returned the systems in either trial phase 1 or trial phase 2. The first trial phase was from April to September 2019. The second phase began in September 2019 and ended in March 2020.

### Study Design

#### Overview

A prospective controlled trial using a wait-list protocol was conducted. To analyze the usage of the *ILSE* app, usage data from the first and second field tests were collected.

**Figure 1:**
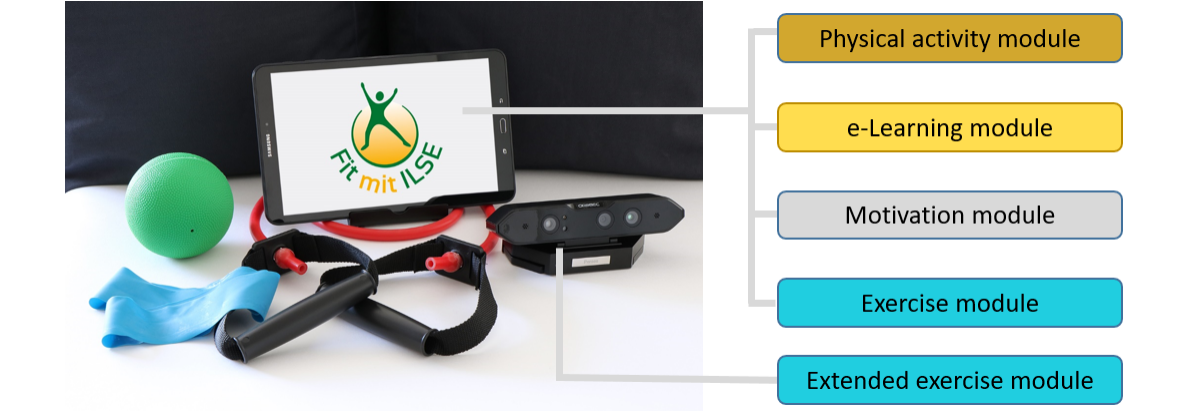
The ILSE app and its modules provided on the tablet version and the 3D camera system version. e-Learning: electronic learning.

The developed system included 2 *ILSE* app versions (1 for the tablet and 1 for the 3D camera system). The app received physical activity data from a fitness tracker (eg, steps, duration, and type of activity) and additional smart home components. The *ILSE* app consisted of 4 different modules for German-speaking participants (see Figure 1 for an overview of the system).

#### Exercise Module

In *Fit zu Hause* (translated as *Fit at home*), the users could access their training plan tailored to their fitness level. Each day, a different workout session of 10, 20, or 30 minutes could be selected, which consisted of exercises to improve strength and balance.

The *ILSE* app version on the 3D camera system, namely the Orbbec Persee [14], supported an overview of the training sessions performed and an advanced exercise module by using skeletal tracking to detect starting positions, counting repetitions, and notifying the trainees when unfavorable positions for selected exercises occurred.

Before the participants received the system, the *ILSE* coaches, who were specifically trained sports scientists, assessed the functional fitness of each participant and configured the functional training plan tailored to the fitness level of the participants.

#### Physical Activity Module

Within *Fit unterwegs* (translated as *Fit on the move*), users could keep an outdoor activity diary by entering their activities manually or by wearing the fitness tracker using the integrated activity recording. Furthermore, a connection to the outdoor platform *outdooractive* [15] enabled users to plan their next outdoor activities (such as hiking or cycling).

#### e-Learning Module

The module *Fit durch Wissen* (translated as *Fit by knowledge*) offered a total of 24 e-learning topics that focused on health- and fitness-promoting exercise training and physical activity for adults ≥55 years old.

#### Motivation Module

The *Erreichtes* (translated as *Achievements*) module displayed the activities performed and the goals achieved in terms of physical activity engagement. The exercise module allowed users to check how much exercise they had performed and the duration of training sessions. In terms of behavior change concepts, all engagements were summarized, and users received weekly medals.

For the following analyses, only those participants who used the *ILSE* app at least once during the field study were included in the analysis. In total, 79% (79/100) of participants from the first test phase and 79% (86/109) of participants in the second test phase used the system. As there were no notable differences in *ILSE* app usage and demographic characteristics between the 2 field test phases [16], the 2 phases were analyzed together to obtain a larger sample size of 165 users.

For each participant, the test week was defined as the week beginning with the day of the week in which they received the system. For example, if they received the system on a Thursday, their test weeks started on Thursdays and ended on Wednesdays. The first test week was assumed to be the familiarization phase for participants; therefore, the first test week was excluded for all. The subsequent analysis of app usage was based on 12 continuous test weeks (test weeks 2-13) for each participant. For the analysis, visits were aggregated per test week and participant.

### Data Collection and Aggregation

Usage data of the *ILSE* app were collected via *Matomo*, an open-source web analytics tool [17]. Usage data describe the log data of the *ILSE* app visits, such as date, time, and visit duration. The main metric of interest for *ILSE* app usage is a *visit*. A visit is defined as the access of a webpage (in our case, the *ILSE* app) by a visitor more than 30 minutes after their last opening of *ILSE* (see the glossary of Matomo for detailed descriptions [17]). For the analysis, this definition of a visit was further narrowed so that it consisted of at least 2 actions within 30 minutes; that is, if someone only opened the app, the visit would not be counted.

To identify the *ILSE* app user types, the analysis focused on those visits that opened the exercise module *Fit at home*. Visits that contained only the other 3 modules were not considered in the following analyses.

Apart from usage data, demographics (age, region, gender, education, and household size) were added to describe app users and find usage patterns. For the analysis, Austrian education levels were grouped according to the International Standard Classification of Education classification [18].

Furthermore, information on fitness level and subjective stated amount of daily exercise and sports was collected at the beginning of the field test to include participants’ previous experience in relation to movement in the analyses. The fitness level as a score between 1 (not fit) and 4 (very fit) was assessed by sports scientists at the beginning of the field test. In addition, participants indicated how much time they spent on average during a typical exercise session. They also indicated how many days per week, on average, they do fitness exercises and how many days they ride a bicycle. Furthermore, the self-reported number of hours they sat on an average day was included in the analysis. As demographic- and exercise-related variables were not available for all participants, Table 1 provides an overview of these variables and the availability of given information based on the number of observations.

**Table 1:** Overview of the demographic- and sports-related variables included in the analysis (N=165).

Characteristics			Observations, n (%)
**Variables**			
	Region	165 (100)	
	Age (years)	165 (100)	
	Gender	165 (100)	
	Household size	165 (100)	
	Education level	165 (100)	
	Fitness level	129 (78.2)	
**Subjective variables**			
	Sports duration	138 (83.6)	
	Number of days with fitness exercises performed	163 (98.8)	
	Hours in sitting position	163 (98.8)	
	Number of days on which they ride a bicycle	78 (47.3)	

### Ethics Approval

All participants provided written informed consent before their participation in the study. The study was conducted in accordance with the guidelines of the Declaration of Helsinki and was approved by the ethics committee of the University of Salzburg (protocol code EK-GZ:09/2018).

### Data Analysis

**Figure 2:**
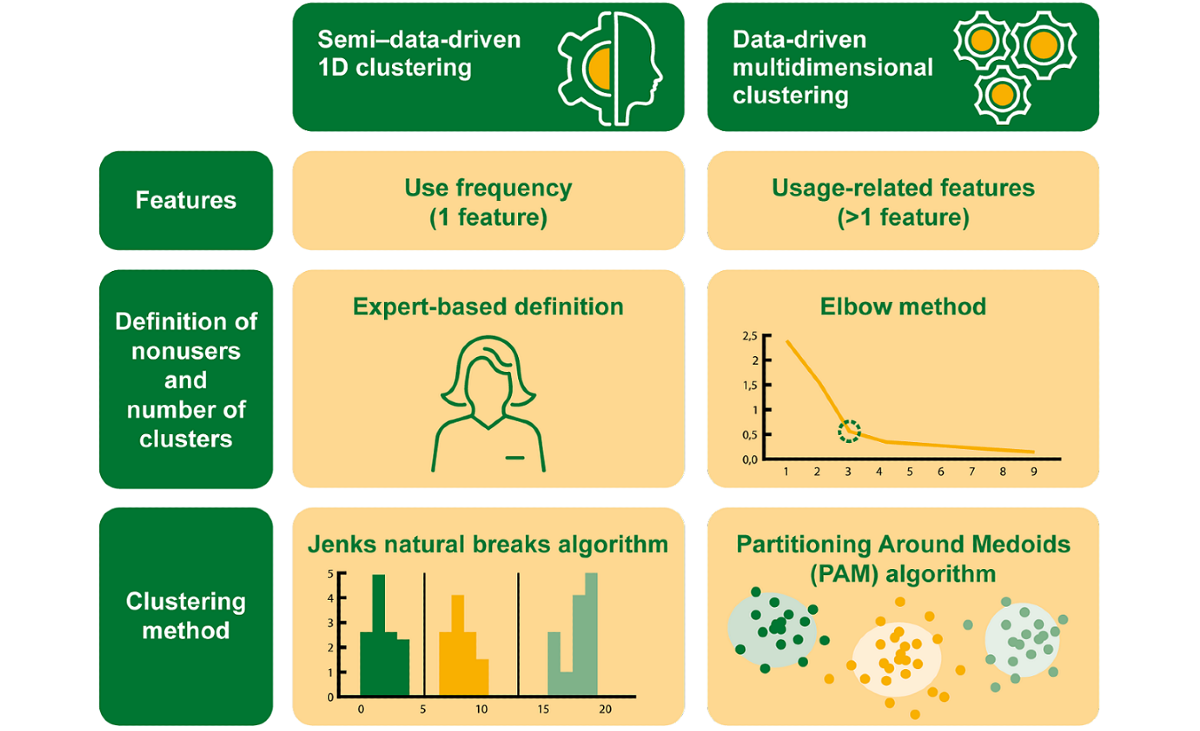
Overview of the 2 user-type clustering approaches.

Statistical analysis was performed using R (R Foundation for Statistical Computing; version 4.1.0) [19]. The clusters based on the 1D algorithm were calculated using the package classInt [20], the multidimensional clustering algorithm with the package cluster [21]. For the generation of plots and data preparation, several packages [22-27] were applied. To test the statistical relationship between app usage types and demographic- and sports-related variables, a chi-square test was applied for categorical variables and a Kruskal-Wallis test was applied for numerical variables. The significance level was set at .05 in both cases. To determine app usage types, 2 data-driven approaches were presented and explored. Figure 2 presents an overview of the 2 clustering approaches.

### Semi–data-Driven Usage Frequency–Based Clustering Approach

This 1D approach is similar to the usual clustering approach for user types, where experts use the usage frequency as a basis to categorize user groups by means of thresholds and, hence, identify subgroups, called clusters, that have cases that are similar to each other but different from other groups in a data set [28]. As in the work of Schneider et al [7], we defined a total of 4 groups but calculated the cutoff points in a data-driven manner with nonequal length based on the Jenks NB [12] clustering algorithm, which identifies the interval thresholds of the groups in a data-driven manner. Jenks NB was developed for the analysis of geographic data and has the additional requirement of a predefined number of clusters [29]. It is similar to the k-means algorithm as it minimizes the within sum of squares of the classes [30].

Furthermore, in the first approach, the lower threshold that defined *low* was set manually. *Low* users were defined as those that did not use the app at least once per week. A single visit was assumed to equate to a training frequency of once per week. There are indications that positive health results can already be expected from a frequency of once per week in the target group of >65 years old [31,32].

### Data-Driven Multidimensional Clustering Approach

As *ILSE* app usage is not only characterized by the pure usage frequency, the second approach took several features into account. The app users were clustered not only by the frequency of the *ILSE* app usage but also by app usage patterns such as *When did they use ILSE?*, *For how many days did they use ILSE?, Did they use the tablet or the camera system?.* Furthermore, at the beginning of the field test, subjective stated number of days per week with fitness exercises performed and the hours in sitting position were included in the multidimensional clustering, as these 2 variables were available for almost all (163/165, 98.8%) users. In total, 14 features were used for the calculations of the multidimensional clusters of 165 users.

As Jenks NB only works for 1D data, a different clustering algorithm needed to be used. We focused on a partitional clustering method because it is easier to interpret and implement than hierarchical approaches [33].

The cluster group size was determined using the Elbow method, and the PAM algorithm (see the study by Kaufman and Rousseeuw [13] for the idea and details of PAM) was applied for cluster calculation as the PAM algorithm is less sensitive to outliers and the sequence of input data compared with k-means [34].

### User Statistics

For the analysis, we included 77% (127/165) women and 23% (38/165) men (compiled from field tests 1 and 2) who used the *Fit at home* function on the tablet or on Orbbec Persee at least once between test weeks 2 and 13. The participants were located in Vienna (79/165, 47.9%) and Salzburg (86/165, 52.1%). They were born between 1946 and 1957 (mean 1952.6, SD 2.36; age ranged from 62-73 years in 2019) and used the *ILSE* app on the tablet, on Orbbec Persee, or on both devices.

## Results

### User Clusters

In the following section, the derived number of cluster groups and cluster thresholds for each approach are presented.

#### Semi–data-Driven Usage Frequency–Based Clustering Approach: Jenks NB

Table 2 lists the cluster thresholds of the 4 user groups based on the application of the Jenks NB algorithm. Applying the expert-based lower threshold for *low* users and the manually set number of 4 groups, 38.2% (63/165) of the participants used the *ILSE* once a week up to every second day. About 30% (49/165) visited the *ILSE* app between once a day and every second day (*moderate* use), and 4% (7/165) used *ILSE* ≥2 times per day (*high* use) on average over the 12 test weeks. Analyzing the descriptive statistics in Table 2 showed that although almost all of the oldest group were *low* or *light* users, the younger users, aged between 62 and 65 years in 2019, generally fell into the *moderate* and *high* user types. In addition, although more than three-fourths (66/86, 77%) of the users from Salzburg were in the *light* or *moderate* group, more than three-fourths (62/79, 78%) of the users living in Vienna were part of the *low* and *light* group. Statistically significant associations were found between the *ILSE* app user-type group and gender (*χ^2^*_3_=9.9; *P*=.02), region (*χ^2^*_3_=13.1; *P*=.004), and age class (*χ^2^*_6_=23.7; *P*<.001). In terms of household size and education, there was no significant relationship between usage group and household size (*χ^2^*_9_=3.1; *P*=.96), as well as education (*χ^2^*_33_=379; *P*=.26).

Table 3 analyzes the app user types with regard to user experience and amount of daily movement. Although 40% (10/24) of the users who were rated as less fit (level 2) are in the *low* usage groups, 19% (7/37) of the very fit participants (level 4) are in the *low* usage group. Furthermore, more users rated as very fit (level 4) or fit (level 3) are in the *moderate* and *high* usage groups than those rated as less fit (level 2). Regarding the self-reported average duration of a training session, it can be shown that those who do <30 minutes of sports activities are in the *low* and *light* usage groups. In addition, at almost 60% (10/17), more people who said they never ride a bicycle are in the *moderate* or *high* usage group than those who said they ride a bicycle frequently (>3 times per week; 5/17, 29%). However, no significant associations between these variables and the app usage group were found (fitness level: *χ^2^*_6_=4.8, *P*=.56; sports duration: *χ^2^*_9_=13.9, *P*=.13; fitness exercises days per week: H_3_=2.1, *P*=.55; sitting hours per week: H_3_=2.8, *P*=.42; days riding a bicycle per week: H_3_=1.9, *P*=.60).

**Table 2:** Demographics of the cluster groups of ILSE app users applying Jenks natural breaks in the semi–data-driven approach. Apart from the birth year, the age reached in 2019 is given in parenthesis (N=165).

Demographics			Low use		Light use		Moderate use		High use
Users, n (%)			46 (27.9)		63 (38.2)		49 (29.7)		7 (4.2)
App visit frequency range (per week)			≤1		1.1-3.6		3.65-7.42		7.49-14.56
**Gender, n (%)**									
	Male	18 (47.4)		10 (26.3)		8 (21.1)		2 (5.2)	
	Female	28 (22)		53 (41.7)		41 (32.3)		5 (3.9)	
**Year of birth (age in years), n (%)**									
	1946-1949 (70-73)	15 (65.2)		7 (30.4)		0 (0)		1 (4.3)	
	1950-1953 (66-69)	18 (25.4)		29 (40.8)		22 (31)		2 (2.8)	
	1953-1957 (62-65)	13 (18.3)		27 (38)		27 (38)		4 (5.6)	
**Location, n (%)**									
	Vienna	30 (38)		32 (40.5)		14 (17.7)		3 (3.8)	
	Salzburg	16 (18.6)		31 (36)		35 (40.7)		4 (4.6)	
**Education, n (%)**									
	ISCED^a^ 2	1 (16.7)		3 (50)		2 (33.3)		0 (0)	
	ISCED 3	14 (21.5)		22 (33.8)		27 (41.5)		2 (3.1)	
	ISCED 4	0 (0)		4 (100)		0 (0)		0 (0)	
	ISCED 5	17 (39.5)		13 (30.2)		10 (23.3)		3 (7)	
	ISCED 6-8	13 (31.7)		18 (43.9)		8 (19.5)		2 (4.9)	
**Type of household, n (%)**									
	Single	11 (26.8)		14 (34.1)		14 (34.1)		2 (4.9)	
	2 persons	29 (26.4)		44 (40)		32 (29.1)		5 (4.5)	
	3-4 person	6 (42.9)		5 (35.7)		3 (21.4)		0 (0)	

^a^ISCED: International Standard Classification of Education.

**Table 3:** Initial training experience grouped to categories and fitness level of the cluster groups of ILSE app users before interventions applying Jenks natural breaks in the semi–data-driven approach (N=165).

		Low use, n (%)	Light use, n (%)	Moderate use, n (%)	High use, n (%)
**Fitness level**					
	2	10 (40)	9 (36)	6 (24)	0 (0)
	3	18 (26.9)	25 (37.3)	20 (29.9)	4 (6)
	4	7 (18.9)	16 (43.2)	11 (29.7)	3 (8.1)
**Number of fitness exercises (per week)**					
	0	13 (38.2)	10 (29.4)	9 (26.5)	2 (5.9)
	1-2	19 (26)	29 (39.7)	23 (31.5)	2 (2.7)
	3-5	12 (26.1)	19 (41.3)	12 (26.1)	3 (6.5)
	6-7	2 (20)	4 (40)	4 (40)	0 (0)
**Duration of sports (minutes)**					
	<30	5 (71.4)	2 (28.6)	0 (0)	0 (0)
	30-60	6 (15.8)	16 (42.1)	14 (36.8)	2 (5.3)
	60-120	12 (17.6)	32 (47.1)	21 (30.9)	3 (4.4)
	>120	8 (32)	9 (36)	7 (28)	1 (4)
**Duration of sitting (hours per week)**					
	<6	17 (26.6)	26 (40.6)	19 (29.7)	2 (3.1)
	6-8	20 (27)	25 (33.7)	26 (35.1)	3 (4.1)
	>8	9 (36)	11 (44)	3 (12)	2 (8)
**Number of days of riding a bicycle (days per week)**					
	0	2 (11.7)	5 (29.4)	9 (52.9)	1 (5.9)
	1-3	13 (29.5)	14 (31.8)	16 (36.4)	1 (2.2)
	>3	4 (23.5)	8 (47.1)	5 (29.4)	0 (0)

#### Data-Driven Multidimensional Clustering Approach: PAM

**Figure 3:**
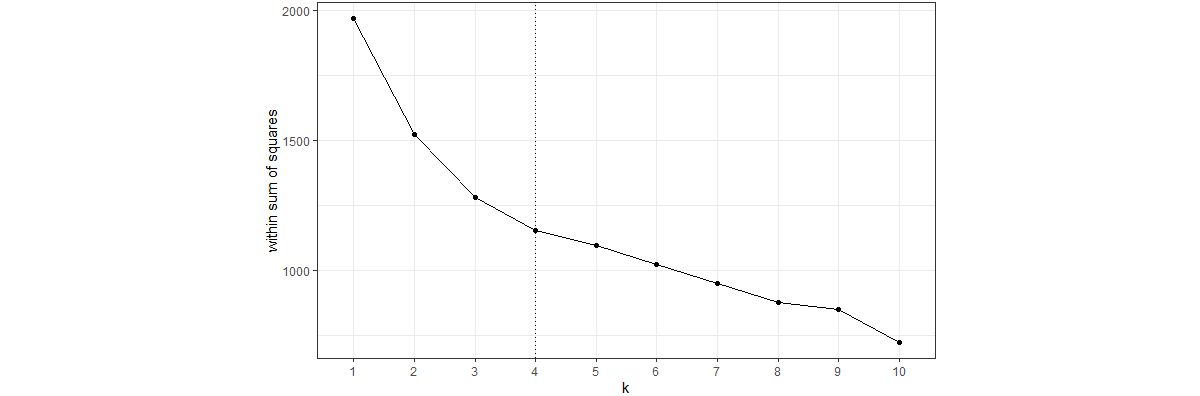
Elbow plot of the multidimensional clustering approach. PAM: Partitioning Around Medoids.

After calculating a number of clusters of size 1 to 10, a total of 4 groups were also used for multidimensional clustering, analogous to the 1D clustering (Figure 3).

**Figure 4:**
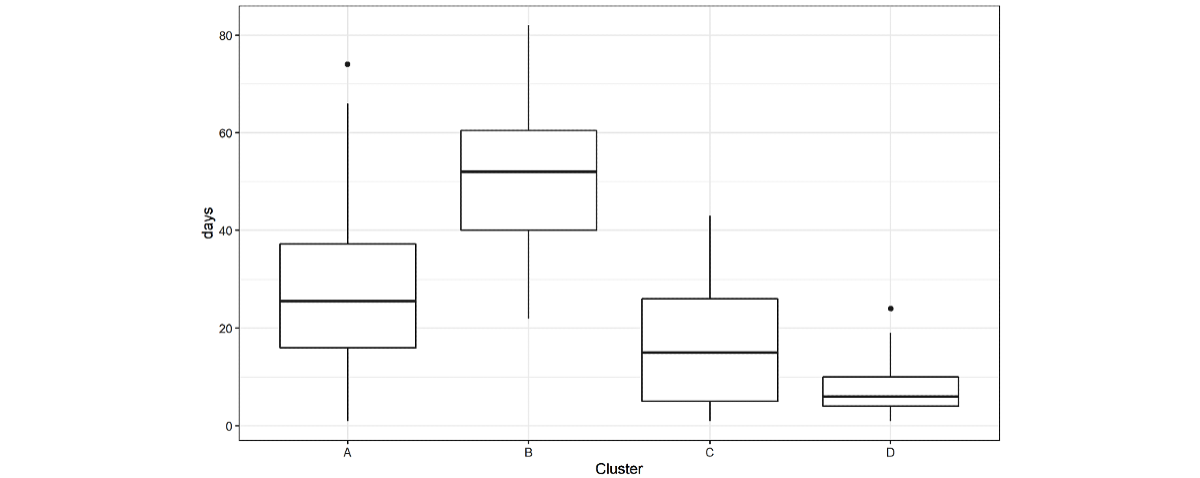
Number of total days of app use in the 4 cluster groups.

Applying the PAM algorithm to the 14 features resulted in 4 clusters of 60 (cluster A), 30 (cluster B), 42 (cluster C), and 33 (cluster D) users. Examination of the clusters showed that cluster B included those users who used the app the most, with a mean use of 50.5 (SD 13.6) total days compared with 8.2 days (SD 5.9) in cluster D (Figure 4).

Table 4 shows the descriptive statistics of the clusters, and Table 5 lists the sports- and fitness-related variables. A detailed analysis of the 4 multidimensional cluster groups revealed the following characteristics.

- Cluster A was formed by users who mainly used the devices in the morning. A total of 80% (48/60) were women, and 55% (33/60) lived in Salzburg.
- Cluster B had the highest number of average app visits and consisted of 90% (27/30) of female users. Two-thirds (20/30, 66%) of users in this cluster lived in Salzburg. *ILSE* app users in this group also made greater use of other features of the *ILSE* app and were younger than those in the other clusters.
- Cluster C was formed by users who used the devices mainly in the evening. A total of 50% (21/42) lived in Vienna, and 50% (21/42) lived in Salzburg.
- Cluster D had the lowest number of average app visits and was formed by 63% (21/33) of users who lived in Vienna and by users who reported doing fitness exercises 4 days per week or less but not more than 4 days. In addition, this cluster was disproportionately formed by men.

**Table 4:** Demographics of the 4 cluster groups derived by the Partitioning Around Medoids algorithm (N=165).

		Cluster A (n=60)	Cluster B (n=30)	Cluster C (n=42)	Cluster D (n=33)
Days of use, mean (SD)		29.0 (16.1)	50.5 (13.6)	15.9 (12.5)	8.2 (5.9)
**Gender, n (%)**					
	Male	12 (20)	3 (10)	10 (23.8)	13 (39.4)
	Female	48 (80)	27 (90)	32 (76.2)	20 (60.6)
**Year of birth (age in years), n (%)**					
	1946-1949 (70-73)	27 (45.5)	10 (33.3)	19 (45.2)	15 (45.5)
	1950-1953 (66-69)	27 (45.5)	10 (33.3)	19 (45.2)	15 (45.5)
	1953-1957 (62-65)	28 (24.2)	20 (66.7)	15 (35.7)	8 (24.2)
**Location, n (%)**					
	Vienna	27 (45)	10 (33.3)	21 (50)	21 (63.6)
	Salzburg	33 (55)	20 (66.7)	21 (50)	12 (36.4)
**Education, n (%)**					
	ISCED^a^ 2	2 (3.5)	1 (3.4)	3 (7.3)	0 (0)
	ISCED 3	24 (42.1)	17 (58.6)	14 (34.1)	20 (31.3)
	ISCED 4	2 (3.5)	0 (0)	1 (2.4)	1 (3.1)
	ISCED 5	13 (22.8)	6 (20.7)	9 (22)	15 (46.9)
	ISCED 6-8	16 (28.1)	5 (17.2)	14 (34.1)	6 (18.8)
**Type of household, n (%)**					
	Single	17 (28.3)	9 (30)	8 (19)	7 (21.2)
	2 persons	36 (60)	20 (66.7)	32 (76.2)	22 (66.7)
	3-4 person	7 (26.7)	1 (3.3)	2 (23.8)	4 (3)

^a^ISCED: International Standard Classification of Education.

**Table 5:** Initial training experience grouped to categories and fitness level of the cluster groups of ILSE app users before interventions applying the Partitioning Around Medoids algorithm (N=165).

			Cluster A (n=60), n (%)		Cluster B (n=30), n (%)		Cluster C (n=42), n (%)		Cluster D (n=33), n (%)
**Fitness level**									
	2	7 (14.3)		2 (8.7)		11 (32.4)		5 (21.7)	
	3	25 (51)		15 (65.2)		17 (50)		10 (43.5)	
	4	17 (34.7)		6 (26.1)		6 (17.6)		8 (34.8)	
**Number of fitness exercises (per week)**									
	0	10 (16.7)		4 (13.3)		9 (4.8)		11 (33.3)	
	1-2	26 (43.3)		16 (53.3)		18 (28.6)		13 (39.4)	
	3-5	16 (26.7)		9 (30)		12 (42.9)		9 (27.3)	
	6-7	7 (11.7)		1 (3.3)		2 (21.4)		0 (0)	
**Duration of sports (minutes)**									
	<30	1 (2)		0 (0)		3 (8.6)		3 (12.5)	
	30-60	16 (31.4)		2 (32.1)		9 (25.7)		4 (16.7)	
	60-120	26 (51)		17 (60.7)		14 (40)		11 (45.8)	
	>120	8 (15.7)		2 (7.1)		9 (25.7)		6 (25)	
**Duration of sitting (hours per week)**									
	<6	19 (32.2)		13 (43.3)		16 (39)		16 (48.5)	
	6-8	33 (55.9)		11 (36.7)		18 (43.9)		12 (36.4)	
	>8	7 (11.9)		6 (20)		7 (17.1)		5 (15.2)	
**Number of days of riding a bicycle (days per week)**									
	0	8 (27.6)		2 (11.8)		7 (41.2)		0 (0)	
	1-3	14 (48.3)		12 (70.6)		8 (47.1)		10 (66.7)	
	>3	7 (24.1)		3 (17.6)		2 (11.8)		5 (33.3)	

## Discussion

### Principal Findings

This study aimed to define *ILSE* app user types and calculate thresholds in a data-driven manner to identify usage patterns. For this purpose, 2 approaches were applied to the usage data of *Fit-mit-*ILSE’s exercise module *Fit at home.* The calculated thresholds within this work were calculated for the specific case of *Fit-mit-ILSE*; therefore, the comparison of the resulting thresholds with other works is impractical. However, the cluster thresholds were compared with the WHO recommendations for functional balance and strength training for adults ≥65 years old (3 or more days per week at moderate or greater intensity) indicates that the *moderate* and *high* user types accomplished the WHO-recommended training frequency. From 1D clustering, 33.9% (56/165) of all users fell into these groups.

Applying the Jenks NB clustering algorithm to the mean app usage per day showed that most of the users of the *Fit-mit-ILSE* program were *light* users of the *ILSE Fit at Home* module, using the module between once a week and every other day with a manual definition of the lowest threshold. The proportion of men in the *low* user group was higher than that of women. In general, male users of the *Fit at home* module were mainly found in the *low* and *light* usage groups. This finding is in contrast to the analysis of Lee [35], who studied 276 older adults from senior centers based on self-administered questionnaire data and found that, in this sample, men engaged in significantly higher amounts of leisure time physical activity than women. Thus, it could be that the *ILSE* app and its functions mainly addressed women, which could be because of several reasons. One assumption could be that the design of the system and the indoor training modules is more likely to motivate women than men, who may prefer to train with physical coaches or independently, without instructions from web-based coaches. As described in the user statistics, 77% (127/165) of the participants were women. This unbalanced sample could influence the analysis of app user groups, as women were more likely to use the app than men, on average. Therefore, future research should include a more gender-balanced sample by, for example, using needs assessments and questionnaires to address men’s expectations related to fitness apps.

Analysis of app user groups in terms of age also showed that older users were disproportionately more likely to be assigned to the *low* and *light* usage groups than younger users, who were more often identified as *moderate* or *high* users. This could be because they are less technology-savvy than younger app users. In the work of Gitlow [36], lack of knowledge, fine motor difficulties, negative attitudes, and age-related physical changes, for example, hearing loss, were identified as barriers for older adults in using technology. To increase older adults’ app usage, additional support could be offered to older participants in the future, particularly concerning technical questions.

In addition, the analysis revealed regional differences in app use, and users living in Salzburg had higher average app use than users from Vienna, suggesting that there may be regional differences between the city of Vienna and the state of Salzburg. Users from these 2 different regions may need to be addressed and motivated differently, which could also be addressed through participatory approaches in the design and development phase of future projects. These results go well with the analysis of Cleland et al [37], who found in their study that adults in rural areas reported significantly more physical activity than adults in urban areas.

As part of the multidimensional clustering approach of *ILSE app* users, 4 different user groups were identified, which differed mainly by the total number of app visits, gender, region, and the time of app use during the day.

### Limitations and Future Research

The analysis of *ILSE* app usage data is subject to some limitations that suggest ideas for future research. Although there was a support team to help with technical and *ILSE* app–orientated questions, some of the users experienced difficulties in using and setting up *ILSE*, especially at the beginning of the field test phases [38]. Therefore, a support team should be planned in future studies, focusing more on supporting older people, as the analysis showed that this group was less likely to use the app.

In addition, there were 3 system issues where the server was shut down for several hours and the *ILSE* app was unavailable during this time, during which use data may be lost. However, as app usage for several weeks was investigated, the shutdown of some hours did not affect the analysis.

Furthermore, as described in the Methods section, the 2 phases of the field test were combined for this analysis. It should be considered that the 2 field tests were conducted in different seasons and that the app was slightly modified (with adapted functions) in the second field test phase. Therefore, the use of the *ILSE* app may be influenced by seasonal effects or seasonal variations. However, as previously reported, there were no remarkable differences in app usage between the 2 phases.

App user types were clustered based on app usage frequency within the study period of 12 weeks. Therefore, changes in usage frequencies over time were not considered and could be interesting for further research.

Participants used *ILSE* in their homes and were instructed to use the app only by themselves. However, owing to the unsupervised setting, other people could theoretically have used the *ILSE* app, which would have increased the number of app visits.

This work focused on analyzing the overall use of the *ILSE* app and focused mainly on the number of visits to the app, as this metric is easy to interpret and comparable with, for example, the WHO recommendations. The correlation between the number of app visits and visit duration per user showed a significantly strong positive Pearson correlation (*r*=0.87). For further studies, however, other data, such as visit duration, should be investigated in detail.

Future research could also investigate the effect of and change in app use frequency on fitness status. A detailed analysis of participants’ dropout reasons and dropouts could also provide relevant insights.

Finally, clustering involves some challenges, such as the definition of the clustering method [39,40]. Another disadvantage of clustering is that different algorithms often result in different partitions [39]. Moreover, determining the number of clusters using the Elbow method is not always clear if the graph has no unique elbow or more than one elbow [41]. Therefore, future work could investigate clustering algorithms other than the Jenks NB and PAM algorithms.

### Conclusions

Applying the Jenks NB and PAM algorithms to the example of *ILSE* app usage data showed that data-driven calculations of user groups can replace expert-based definitions and provide objective thresholds for the analysis of app usage data.

For example, when evaluating a new fitness app, statistical methods and data-driven clustering techniques could help identify the impact of this newly developed app on subgroups of a particular population, describe usage patterns and users, and draw conclusions about which groups of people may not yet be addressed. Using these insights, groups that have not yet been targeted can be specifically addressed and supported.

The cluster analysis of the *Fit at home* module of the *ILSE* app revealed differences in app use by gender, age, region, and time of app use. On average, higher app use was observed among women, younger users, and app users living in Salzburg.

In general, from determining the number of cluster groups to identifying cluster thresholds and ranges, data science offers a variety of alternative methods and algorithms to identify patterns in data.

## Abbreviations

AAL: active and assisted living
Jenks NB: Jenks natural break
PAM: Partitioning Around Medoids
WHO: World Health Organization
